# The Impact of Litter from Different Belowground Organs of *Phragmites australis* on Microbial-Mediated Soil Organic Carbon Accumulation in a Lacustrine Wetland

**DOI:** 10.3390/microorganisms13051146

**Published:** 2025-05-16

**Authors:** Chong Chen, Yong Wang, Liu Yang, Yongen Min, Keming Yue, Sitong Lu, Hongfeng Bian, Xue Wang, Leilei Zhang

**Affiliations:** Key Laboratory of Wetland Ecology and Vegetation Restoration, Ministry of Ecology and Environment, Northeast Normal University, Changchun 130117, China; chenc282@nenu.edu.cn (C.C.); yangl752@nenu.edu.cn (L.Y.); minye955@nenu.edu.cn (Y.M.); yuekm923@nenu.edu.cn (K.Y.); lust366@nenu.edu.cn (S.L.); wangx881@nenu.edu.cn (X.W.); zhangll554@nenu.edu.cn (L.Z.)

**Keywords:** belowground plant litter, community assembly, soil microorganisms, soil organic carbon, survival strategies

## Abstract

Although belowground litter decomposition critically influences lacustrine wetland soil carbon dynamics, the organ-specific microbial mechanisms driving soil organic carbon (SOC) accumulation remain unclear. Existing research has predominantly focused on aboveground litter, leaving a significant gap in the understanding of how roots and rhizomes differentially regulate carbon cycling through microbial community assembly and survival strategies. This study took *Phragmites australis* (a plant characteristic of lacustrine wetland) as the research object and examined how decomposing belowground litter from different organs affects microbial-mediated SOC accumulation through a one-year in situ field incubation in Jingyuetan National Forest Park, Changchun City, Jilin Province, China. Our findings reveal that root litter exhibited the highest decomposition rate, which was accelerated by intermittent flooding, reaching up to 1.86 times that of rhizome. This process enriched r-strategist microbial taxa, intensified homogeneous selection, and expanded niche width, directly promoting SOC accumulation. Rhizome litter decomposition enhanced dispersal limitation, promoted K-strategist microbial dominance, and indirectly modulated SOC through soil acidification. Mixed-litter treatments significantly enhanced SOC accumulation (up to three times higher than single-litter treatments) through synergistic nutrient release (non-additive effects < 0.04) and reinforced microbial network interactions. SOC accumulation varied significantly with the flooding regime as follows: non-flooded > intermittent flooding > permanent flooding. This study provides new insights into the microbially driven mechanisms of plant-organ-specific decomposition in the carbon cycling of wetland ecosystems.

## 1. Introduction

Wetlands represent some of Earth’s most ecologically significant ecosystems, serving as critical hubs for biodiversity conservation and as providers of essential ecosystem services [1,2]. As key components of these ecosystems, wetland soils function as massive organic carbon reservoirs, playing a large role in global carbon cycling. Remarkably, while occupying merely 6% of the planet’s terrestrial surface [3,4], wetlands sequester an estimated 20–30% of the world’s soil carbon stock. This carbon storage capacity highlights their unparalleled importance in regulating global carbon budgets and mitigating climate change.

Litter decomposition serves as a fundamental biogeochemical process that regulates soil carbon pool dynamics in lacustrine wetland ecosystems, where emergent macrophytes (e.g., *Phragmites australis* (Cav.) Trin. ex Steud.) and submerged vegetation dominate plant communities and produce distinct litter types [5]. The existing literature broadly categorizes litter quality based on biochemical composition, with “low-quality” litter characterized by high C/N, a trait that is particularly pronounced in the belowground organs of lacustrine wetland sedges and rushes. Meanwhile, “high-quality” litter exhibits low ratios of these parameters [6]. Although the effects of aboveground litter on soil organic carbon (SOC) have been extensively studied, substantial discrepancies persist in the reported findings: Córdova et al. reported greater SOC accumulation with “high-quality” litter inputs in lacustrine wetlands with tidal flushing [7], whereas Lyu et al. demonstrated superior SOC stabilization with “low-quality” litter [8]. Other studies have found no significant differences among litter types [9]. Notably, belowground root-derived litter contributes substantially to terrestrial carbon cycling, representing up to 48% of total plant litter inputs, exceeding the contributions from leaf fall (41%) and stem litter (11%) in many ecosystems [10]. Belowground plant organs intimately interact with soil matrices, serving as direct sources of soil carbon. Root-derived carbon inputs influence the dynamics and stability of soil carbon pools by facilitating the formation of soil organic carbon [11]. The input of belowground litter provides additional labile carbon substrates that stimulate microbial activity and accelerate the decomposition of older soil organic carbon through the “priming effect”, thereby modifying soil carbon pool dynamics [12]. Due to diverse legacy effects [13,14] associated with the litter traits of different belowground organs, decomposition rates vary substantially among organ types. This variability in decomposition kinetics may introduce uncertainty in assessing the impacts of belowground plant litter on lacustrine wetland soils [15]. However, at present, research regarding both the decomposition processes of belowground plant litter under fluctuating water level regimes characteristic of lacustrine wetlands and the differential impacts of litter quality on soil organic carbon dynamics remains insufficient. Moreover, under natural conditions, the co-decomposition of aboveground litter with varying “quality” generates non-additive effects, specifically synergistic or antagonistic interactions [16] that can exert either positive or negative influences on SOC. However, the non-additive effects resulting from the mixed decomposition of belowground plant organs with differing “quality”, along with their subsequent impacts on soil organic carbon in lacustrine wetlands, remain poorly understood.

Soil microorganisms are recognized as pivotal drivers of global biogeochemical cycles [17], while plant litter serves as the fundamental substrate for microbial growth and metabolism [18]. Esperschutz et al. proposed that during litter decomposition, microorganisms directly participate in the process, facilitating the breakdown of organic carbon and the transformation of nutrients. The release of nutrients from decomposing litter creates favorable growth conditions for microbial communities, thereby altering their composition, which, in turn, influence subsequent decomposition dynamics [19]. Different microbial taxa possess distinct metabolic pathways: bacterial groups exhibit a specialized capacity for the rapid decomposition of litter components rich in simple sugars and proteins [20], whereas fungal communities demonstrate unique enzymatic advantages in degrading recalcitrant compounds, such as lignin and cellulose [21,22]. Research has demonstrated that “high-quality” litter induces microbial communities to adopt r-strategies, whereas “low-quality” litter favors K-strategies, enhancing resource acquisition efficiency through mycelial network expansion and interspecific metabolic complementarity [23]. Studies on microbial community assembly processes play a pivotal role in elucidating the underlying mechanisms governing microbial diversity, compositional dynamics, and ecosystem functioning [24,25]. The assembly of microbial communities is primarily influenced by two complementary mechanisms: deterministic processes and stochastic processes [26]. Deterministic processes are based on niche theory, shaping specific niches for species with unique traits to control community composition and species abundance, involving the regulation of abiotic environmental selection and various biotic interactions [27]. Stochastic processes are based on neutral theory, assuming a stochastic balance between species loss and gain, with all species possessing the same niche [28]. Studies have revealed that litter decomposition directly and indirectly regulates forest soil biotic communities, thereby influencing soil ecological processes [29]. Litter inputs of differing quality create heterogeneous resource availability and habitat conditions, which can significantly alter soil pH, nutrient availability, and organic matter content. These physicochemical modifications ultimately determine the reproductive success and habitat suitability for specific microbial taxa, thereby shaping their spatial distribution and community assembly patterns [30]. Research has revealed that “high-quality” litter strengthens environmental filtering to increase the dominance of deterministic processes, driving community convergence toward specific functional groups. The resulting homogenized environment intensifies interspecies competition and promotes competitive exclusion. In contrast, “low-quality” litter, through increasing the difficulty of resource acquisition, may enhance the contribution of stochastic processes through dispersal limitation and random extinction events, while the resulting heterogeneous microhabitats improve species coexistence potential [31]. Most studies on how litter affects microbial survival strategies and community assembly have focused on aboveground litter, with only a minority addressing root litter. However, many wetland plants possess not only roots but also modified underground organs, such as rhizomes. The effects of root versus rhizome litter, either separately or in combination, on microbial communities and their subsequent impacts on soil organic carbon dynamics have yet to receive widespread research attention.

In lacustrine wetland ecosystems, *P. australis*, as a typical rhizomatous species, exhibits remarkable belowground organ heterogeneity; its robust rhizomes constitute “low-quality” litter, while the well-developed roots represent “high-quality” litter. Flooding, as a key environmental factor in wetland ecosystems, profoundly regulates organic matter transformation processes by controlling redox conditions. Permanently flooded, anaerobic conditions significantly suppress microbial activity, slowing litter decomposition rates and promoting organic matter accumulation. In contrast, non-flooded conditions create aerobic environments that enhance microbial metabolism; although this accelerates litter decomposition, it may also result in organic carbon loss via mineralization [32]. The current understanding of the mechanisms by which belowground organic litter influences soil organic carbon pool dynamics and regulates microbial survival strategies remains limited. Resolving this knowledge gap is crucial for understanding the carbon cycling processes driven by litter–soil–microbe interactions in lake wetland ecosystems. In this study, *P. australis* litter was used to explore how belowground organic litter decomposition influences soil organic carbon pools and microbial survival strategies in a flooded environment. We propose the following hypotheses: (1) the addition of belowground organ litter alters SOC pools, with “high-quality” root litter exhibiting more pronounced effects; and (2) the decomposition of distinct belowground organ induces divergent shifts in microbial community structure in soil, where “high-quality” root litter promotes a transition toward r-strategist microbial taxa and enhances deterministic processes in community assembly, thereby influencing SOC accumulation.

## 2. Materials and Methods

### 2.1. Study Area

**Figure 1:**
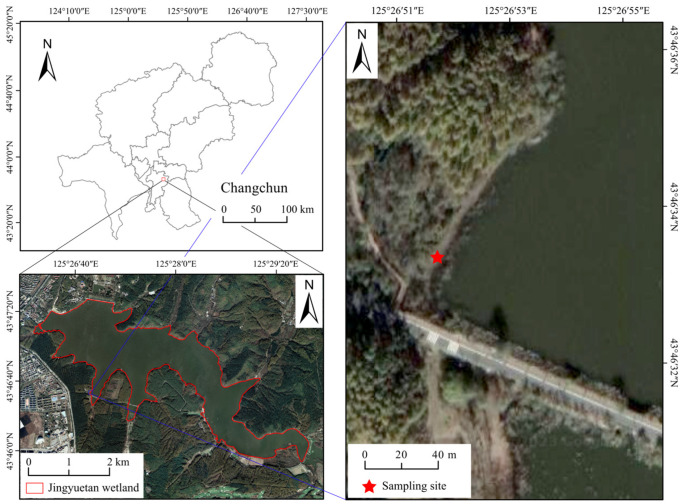
Maps of the sample plot in Jingyuetan National Forest Park in Changchun City, Jilin Province.

The study site is located in Jingyuetan National Forest Park, Changchun City, Jilin Province, China (Figure 1). This park represents a transitional zone between the Changbai Mountain foothills and the western grasslands and is situated within a temperate semi-humid monsoon climate region. The water level of Jingyuetan Lake fluctuates, with elevations between 233.0 and 234.0 m. The lake is primarily recharged by rainfall, with excess water discharging through the overflow dam into the Xiaoheyanzi River when the water level reaches 234.0 m. The soil temperature at Changchun Jingyuetan National Forest Park from November 2022 to November 2023 showed a summer high and winter low pattern (Figure S1). Similar conclusions can be drawn from the historical monthly temperature trend records of Changchun Jingyuetan. For instance, the lowest temperature (−28 °C) occurred in February, while the highest temperature (34 °C) was recorded in July. Precipitation also followed a similar fluctuation pattern, with the highest rainfall (61.7 mm) in July and the lowest (0.1 mm) in December. These data were sourced from the Jilin Provincial Meteorological Bureau (http://jl.cma.gov.cn/, accessed on 15 November 2023). These precipitation patterns directly lead to water level fluctuations, such as intermittent flooding. *P. australis* is a dominant riparian plant species around the lake, characterized by horizontally growing rhizomes containing aerenchyma tissue and rich reserves of starch, sugars, lignin, and defensive secondary metabolites (e.g., phenolics, alkaloids). In contrast, its roots exhibit slender morphology with abundant soluble sugars, amino acids, and nutrient-absorption-related transporter proteins [33].

### 2.2. Sample Preparation

In early October 2022, before complete plant senescence, we established three 1 m × 1 m × 1 m quadrats in *P. australis*-dominated communities (*P. australis* account for up to 95% of the individuals, with only sparse occurrences of herbaceous plant species observed, such as *Alisma plantago-aquatica*, *Echinochloa crus-galli*, and so on.) to collect belowground plant organs. After drying at 65 °C to a constant weight, the average belowground biomass was determined to be 6 g/m^3^ (dry biomass), with a biomass ratio of roots to rhizomes at 1:2. The roots were cut into 10 cm segments and the rhizomes into 2 cm pieces [19]. For the decomposition experiment, we weighed out 6 g/m^3^ each of rhizome litter (single species), root litter (single species), and mixed rhizome–root litter. These samples were then placed in 15 × 25 cm litter bags with a 5 mm upper mesh and a 2 mm lower mesh [34] for field incubation.

*P. australis* was distributed across elevations ranging from 233.0 to 234.1 m. Study transects were established at three representative elevations: 233.0 m (HI, permanently flooded), 233.5 m (ME, intermittently flooded), and 234.1 m (LO, non-flooded). Four treatments were applied with four replicates each: root litter (LR), rhizome litter (L), mixed litter (M, roots: rhizomes = 1:2 ratio), and no litter addition control (CK). The experiment was initiated in November 2022 (early winter). All surface litter was removed before setup (to avoid interference from fallen objects on the ground and keep the underground conditions consistent). To minimize cross-treatment interference, the litter bags were spaced 1 m apart within each transect and buried at a 15 cm depth after thorough mixing with soil. The litter bags were placed under the soil where reeds originally grew [35]. Following a full year of decomposition (until November 2023), both the remaining litter and surrounding soil samples were collected for laboratory analysis. Simultaneously, we installed three button-shaped thermometers at the same soil depth to record the soil temperature from November 2022 to November 2023.

### 2.3. Measurement Methods

Using forceps, the decomposed belowground organs were carefully collected onto sieves and rinsed with ultrapure water. The samples were then oven-dried at 65 °C and weighed. Subsequently, a grinding mill was used to homogenize the plant material through a 100-mesh sieve for subsequent chemical analysis. The soil samples were passed through a 2 mm sieve to remove debris and then divided into three aliquots. The first aliquot was air-dried and ground through a 100-mesh sieve for analysis of the SOC, total nitrogen (TN), available phosphorus (AP), dissolved organic carbon (DOC), and pH. The second aliquot was freeze-dried and ground through a 100-mesh sieve for phospholipid fatty acid (PLFA) analysis of the microbial biomass. The third aliquot was stored at −80 °C for microbial DNA extraction, PCR amplification, and sequencing.

#### 2.3.1. Determination of Plant Basic Parameters

The litter was oven-dried at 65 °C and weighed. The ground and sieved litter samples were analyzed for carbon (C) and nitrogen (N) content using an elemental analyzer (Vario MACRO Cube, Langenselbold, Germany). The litter mass loss, decomposition rates, and non-additive effects of the mixed litter were calculated according to the following formulas [16]:

(1) Mass Loss (R):

(1) $$R=\frac{M_{t0}-M_{t}}{M_{t0}}\times 100\%$$

where M_t0_ is the initial dry mass of the litter, and M_t_ is the remaining dry mass of the litter at time t.

(2) Decomposition Rate (SL):

(2) $$\mathrm{SL}=\frac{M_{t1}-M_{t2}}{t_{2}-t_{1}}\times 100\%$$

where M_t1_ is the dry mass of the litter at time t_1_, and M_t2_ is the remaining dry mass of the litter at time t_2_.

(3) Non-Additive Effects (YME):

(3) $$\mathrm{Expected}\;\mathrm{YML}\;\left(\%\right)=\frac{M_{\mathrm{ta}}X_{\mathrm{ta}}Y_{\mathrm{ta}}+M_{\mathrm{tb}}X_{\mathrm{tb}}Y_{\mathrm{tb}}}{M_{\mathrm{ta}}X_{\mathrm{ta}}+M_{\mathrm{tb}}X_{\mathrm{tb}}}$$

(4) $$\mathrm{YME}=\frac{\mathrm{Observed}\;\mathrm{YML}-\mathrm{Expected}\;\mathrm{YML}}{\mathrm{Expected}\;\mathrm{YML}}$$

where Y_ta_ and Y_tb_ are the nutrient retention rates (%) of the two types of plant litter when decomposed separately. M_ta_ and M_tb_ are the initial masses of the two types of plants (a and b) in the mixture. X_ta_ and X_tb_ are the initial nutrient contents of the two types of plants (a and b) in the mixture. The observed YML (Y represents the nutrient contents of C and N) is the actual mass retention rate of the mixed litter. The expected YML is the expected nutrient retention rate of the mixed litter under ideal conditions. YME is the mixture effect of the litter (YME > 0 indicates antagonism, with more nutrients retained in the litter and fewer nutrients released into the soil; YME < 0 indicates synergism, with fewer nutrients retained in the litter and more nutrients released into the soil).

#### 2.3.2. Determination of Soil Basic Parameters

The soil pH was measured in a 1:1.25 soil–water suspension using a pH meter (Mettler-Toledo, Zurich, Switzerland). The SOC and TN contents were determined using an elemental analyzer (Vario MACRO cube, Langenselbold, Germany). The DOC was extracted with K_2_SO_4_ (soil: extractant = 1:5 *w*/*v*), filtered through a 0.45 μm membrane, and analyzed using a TOC analyzer (Shimadzu, Kyoto, Japan). The soil AP content was measured using the molybdate–ascorbic acid method.

#### 2.3.3. Determination of Soil Microbial Biomass

The soil microbial biomass and community composition were assessed using PLFA analysis [36]. Briefly, PLFAs were extracted from 5 g of freeze-dried soil. The extracted fatty acid methyl esters (FAMEs) were redissolved in hexane containing 19:0 as an internal standard and subsequently detected using gas chromatography–mass spectrometry (Agilent 7890A, Santa Clara, CA, USA). Following fatty acid quantification, biomarkers with relative abundances < 0.5% were excluded from the analysis.

#### 2.3.4. Bacterial and Fungal Community Structure

Microbial community structure changes were determined using 16S rRNA and ITS high-throughput sequencing. Genomic DNA was extracted from the soil samples. Bacterial diversity was analyzed using primers 341F (CCTAYGGGRBGCASCAG) and 806R (GGACTACNNGGGTATCTAAT), targeting the V3-V4 region of bacterial 16S rDNA. Fungal diversity was analyzed using primers ITS1-1F-F (CTTGGTCATTTAGAGGAAGTAA) and ITS1-1F-R (GCTGCGTICTICATCGATGC), targeting the ITS1 region. PCR amplification was performed as follows: the initial denaturation was carried out at 98 °C for 1 min, followed by 30 cycles of 98 °C for 10 s, 50 °C for 30 s, and 72 °C for 30 s, with a final extension at 72 °C for 5 min. The PCR products were pooled, purified, and examined using electrophoresis on 2% agarose gels. The target fragments were recovered using a DNA purification kit (Tiangen Biotech, Beijing, China). Library preparation included end repair, A-tailing, adapter ligation, and purification. The quality of the library was verified using AATI and qPCR. Sequencing was performed on the Illumina NovaSeq platform (Novogene Bioinformatics Technology Co., Ltd., Beijing, China).

### 2.4. Statistical Analysis

For the soil physicochemical properties and microbial biomass data, descriptive statistics were used to characterize the basic data features. The normality of the data was assessed using the Shapiro–Wilk test (*p* > 0.05). Homogeneity of variances was confirmed via Levene’s test (*p* > 0.05). For variables violating assumptions (e.g., non-normal residuals or unequal variances), robust statistical methods (e.g., Welch’s ANOVA or non-parametric tests) were applied (Tables S1–S4). Meanwhile, one-way ANOVA with Tukey’s post-hoc test was performed to assess significant differences among the treatment groups. For both the bacterial (16S rRNA gene sequencing) and fungal (ITS sequencing) community data, we followed parallel analytical workflows. After data preprocessing, we conducted α-diversity analyses to assess community richness and diversity and β-diversity analyses to compare structural differences, and we performed in-depth investigations through community composition analysis, network analysis, niche breadth calculation, neutral community model (NCM) testing, and βNTI analysis to examine the community assembly, interaction patterns, and the relative importance of deterministic versus stochastic processes. The analytical tools included multiple software platforms: R 4.2.2 (packages: vegan, phyloseq, picante, igraph), QIIME2 2023.2, SPSS 20, and GraphPad Prism 10, along with visualization tools (ggplot2, ggpubr, Circos, and Gephi). Partial least squares path modeling (PLS-PM) was performed using the “plspm” package in R 4.2.2 [37]. The model was refined based on significance (*p* < 0.05) and goodness-of-fit (GOF > 0.6) in order to determine how litter decomposition dynamics influence the soil organic carbon pool through microbial mediation.

## 3. Results

### 3.1. Litter Decomposition Dynamics

**Figure 2:**
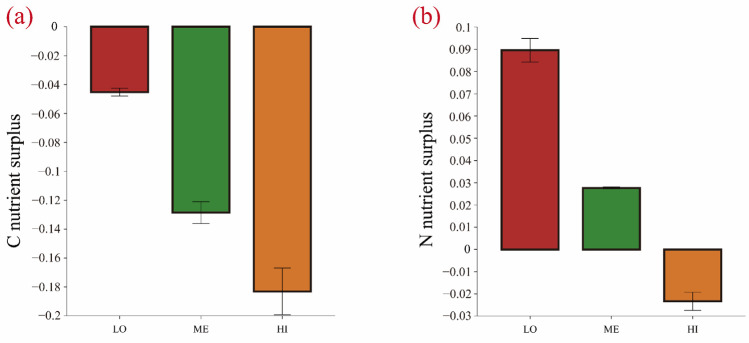
Non-additive effects of mixed litter. (**a**) Litter C nutrients remaining, (**b**) litter N nutrients remaining. LO indicates non-flooded, ME indicates intermittently flooded, and HI indicates permanently flooded. The standard error is used to represent the error bar in the figure.

Regardless of the flooding conditions, LR exhibited the fastest decomposition rate, while L showed the slowest. The decomposition rate of the roots reached up to 1.28–1.86 times that of the rhizomes. The patterns of the litter mass loss and decomposition rates followed ME > HI > LO. Under ME conditions, the litter mass loss and decomposition rates were 1.29–1.38 times and 1.59–1.99 times higher than those under LO conditions, respectively. Compared to L litter, LR litter displayed a lower C content, higher N content, and a lower C/N ratio. Litter under non-flooded conditions exhibited higher C and N contents compared to the other two flooding regimes (Table 1). For the mixed litter, non-additive effects on residual C showed synergistic interactions, while residual N displayed synergistic effects under permanently flooded conditions but antagonistic effects under non-flooded and intermittently flooded regimes (Figure 2).

### 3.2. Effects of Litter Input on Soil Organic Carbon and Physicochemical Properties

Overall, the M treatment yielded the highest SOC content (8.50–25.99 g/kg), while the L treatment resulted in the lowest (7.11–18.39 g/kg), with the DOC following a similar pattern. For TN under the LO and ME conditions, the order was LR > L > M > CK, whereas under the HI conditions, it became M > LR > L > CK. Different litter addition methods variably affected the soil AP. The soil SOC, DOC, TN, and AP contents consistently showed a pattern of LO > ME > HI. The initial soil pH ranged from 6.5 to 7.1, which litter addition generally reduced (Table 2).

### 3.3. Effects of Litter Input on Soil Microbial Biomass

**Figure 3:**
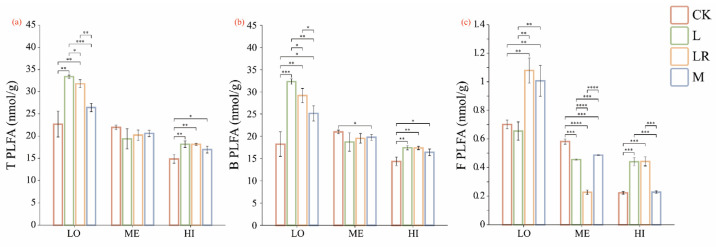
Changes in soil (**a**) T PLFA (total microbial biomass), (**b**) B PLFA (bacterial microbial biomass), and (**c**) F PLFA (fungal microbial biomass) under different flooding conditions after litter decomposition. LO indicates non-flooded, ME indicates intermittently flooded, HI indicates permanently flooded, CK represents the blank control without adding litter, L represents rhizomes, LR represents roots, and M represents a mixture of the two. * *p* < 0.05, ** *p* < 0.01, *** *p* < 0.001, **** *p* < 0.0001.

Under identical flooding conditions, different litter addition treatments exhibited distinct effects on the total PLFA (T PLFA), bacterial PLFA (B PLFA), and fungal PLFA (F PLFA). The L treatment demonstrated the highest T PLFA and B PLFA contents, whereas the LR treatment displayed the highest F PLFA content. The patterns of T PLFA, B PLFA, and F PLFA across the flooding regimes also varied, consistently showing LO > ME > HI (Figure 3).

### 3.4. Effects of Litter Input on Soil Microbial Community Characteristics

In this study, high-throughput sequencing was used to analyze the species distribution of soil bacteria and fungi. The Chao1 index [38] and the Shannon index [39] were used as indicators for the analysis of soil bacterial alpha diversity and richness.

**Figure 4:**
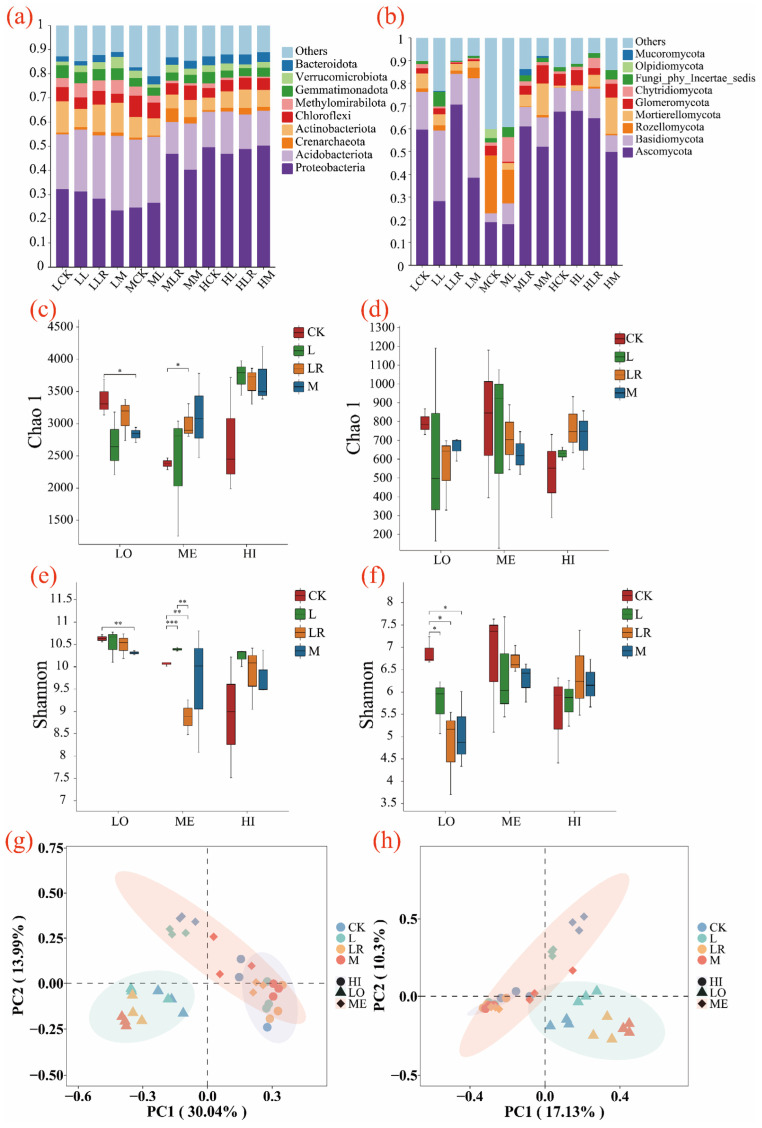
Soil microbial characteristics after litter decomposition under different flooding conditions: (**a**) bacterial and (**b**) fungal community relative abundance; α-diversity differences in (**c**,**e**) bacteria and (**d**,**f**) fungi across flooding conditions and litter addition methods. PCoA analysis based on Bray–Curtis distance showing (**g**) bacterial and (**h**) fungal community composition under varying flooding conditions and litter addition methods. LO indicates non-flooded, ME indicates intermittently flooded, HI indicates permanently flooded, CK represents the blank control without adding litter, L represents rhizomes, LR represents roots, and M represents a mixture of the two. * *p* < 0.05, ** *p* < 0.01, *** *p* < 0.001.

Following the litter addition treatments, the predominant bacterial phyla in the soils were Proteobacteria and Acidobacteriota. Overall, the LR treatment resulted in the highest relative abundance of Proteobacteria and the lowest relative abundance of Acidobacteriota compared to the L and M treatments. After one year of litter decomposition, the soils receiving the LR treatment exhibited the highest microbial α-diversity indices, while those with the L treatment showed the lowest. The soil fungal communities following the litter addition treatments were dominated by Ascomycota and Basidiomycota. Overall, the LR treatment showed the highest relative abundance of Ascomycota and the lowest relative abundance of Basidiomycota compared to the L and M treatments. After one year of litter decomposition, the LR-treated soils exhibited the highest microbial α-diversity indices, while the L-treated soils showed the lowest. Principal coordinates analysis (PCoA) based on Bray–Curtis distances revealed differences in soil microbial community structure separation. For both bacterial and fungal communities, significant differences in community composition and diversity were observed among the different litter treatments under the LO and ME conditions, whereas the HI conditions did not significantly alter microbial β-diversity (Figure 4).

**Figure 5:**
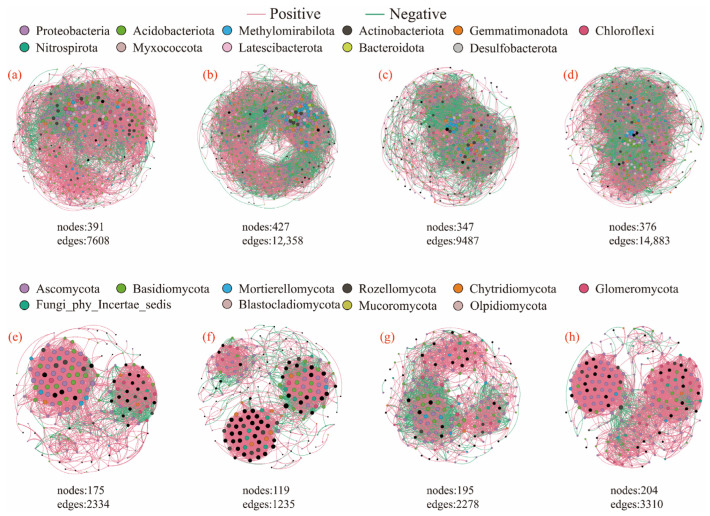
Soil microbial network analysis after different litter decomposition treatments: (**a**–**d**) bacterial and (**e**–**h**) fungal communities. (**a**,**e**) Control (no litter), (**b**,**f**) rhizome litter, (**c**,**g**) root litter, and (**d**,**h**) mixed litter treatments.

Network analysis revealed that litter addition substantially enhanced microbial interactions. For both the bacterial and fungal communities, the litter treatments significantly increased microbial connectivity. All three litter treatments elevated negative connections, average clustering coefficients, and mean degrees. Furthermore, LR addition increased the modularity coefficient in the fungal communities and extended the average path length in the bacterial networks (Figure 5 and Table 3).

**Figure 6:**
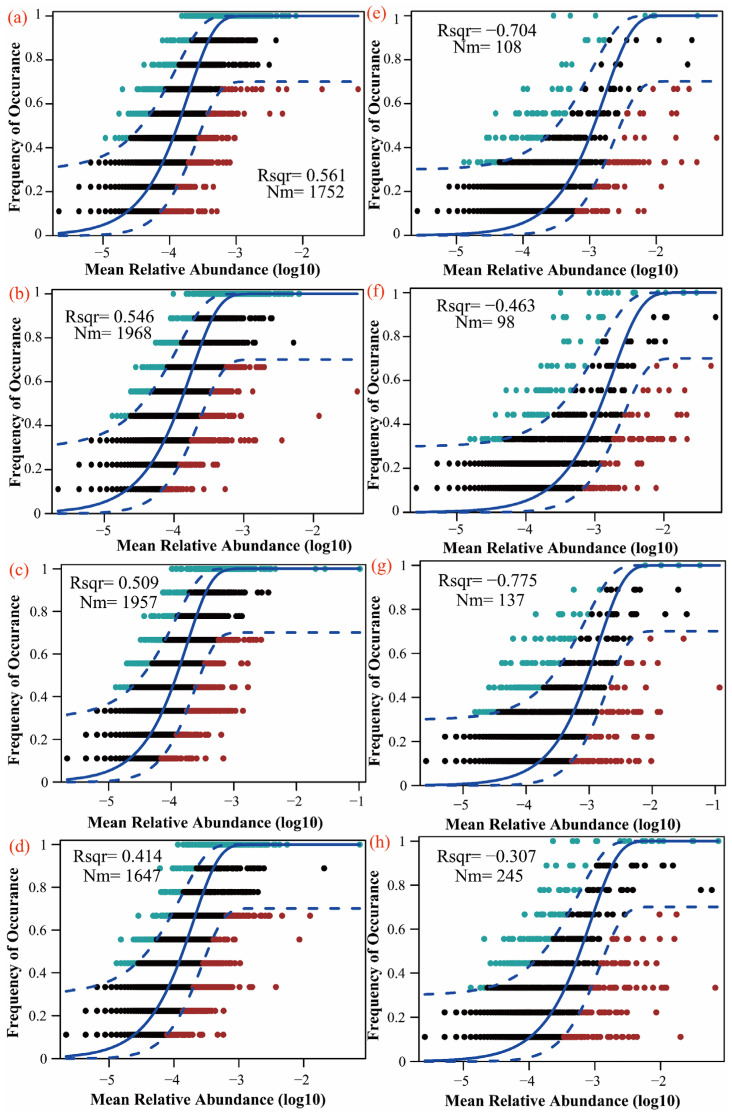
Neutral community model (NCM) analysis of soil (**a**–**d**) bacterial and (**e**–**h**) fungal communities following different litter decomposition treatments: (**a**,**e**) control (no litter), (**b**,**f**) rhizome litter, (**c**,**g**) root litter, and (**d**,**h**) mixed litter treatments. The solid blue line represents the best-fit values of the neutral community model, the dashed blue line represents the 95% confidence interval of the model (estimated through 999 bootstraps), and OTUs with occurrence frequencies higher or lower than predicted by the neutral community model are displayed in different colors.

**Figure 7:**
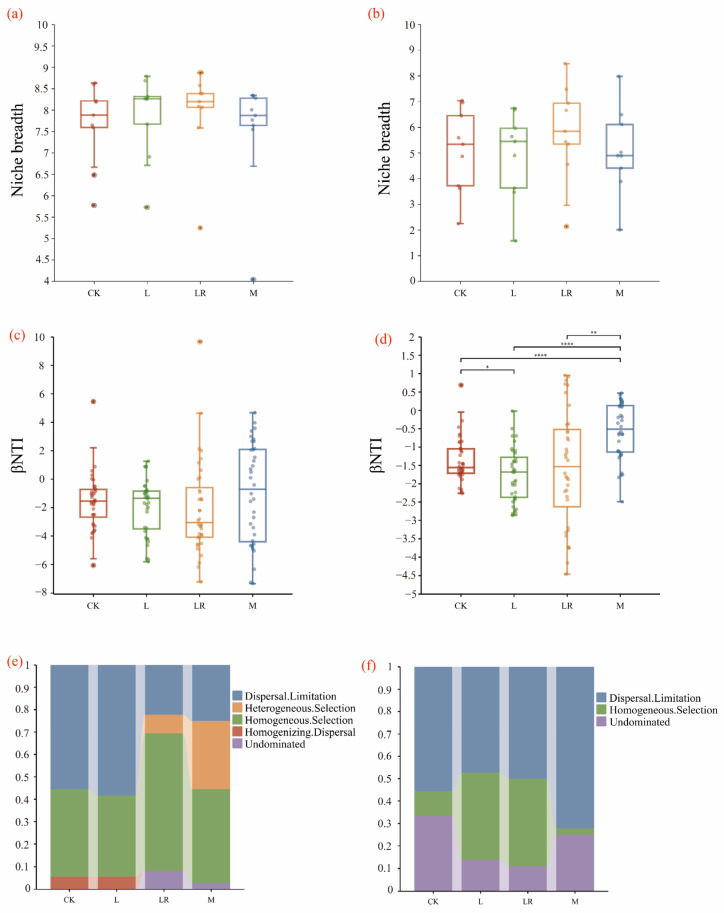
Community assembly characteristics of bacteria and fungi after litter decomposition. Niche breadth of (**a**) bacteria and (**b**) fungi; βNTI distributions of (**c**) bacterial and (**d**) fungal communities; proportional contributions of assembly processes for (**e**) bacterial and (**f**) fungal communities. CK represents the blank control without adding litter, L represents rhizomes, LR represents roots, and M represents a mixture of the two. * *p* < 0.05, ** *p* < 0.01, **** *p* < 0.0001.

The neutral community model (NCM) estimated the relationship between OTU occurrence frequency and relative abundance changes. The NCM only explained bacterial community structure changes following litter addition (R^2^ > 0). Both the L (Nm = 1968) and LR (Nm = 1957) treatments showed higher Nm values than CK (Nm = 1752) and M (Nm = 1647), indicating greater microbial species dispersal in the L- and LR-treated soils compared to those treated with CK and M (Figure 6). βNTI was used to quantify phylogenetic turnover among the different litter treatments. For the bacterial communities, litter addition increased homogeneous selection and decreased dispersal limitation, with the LR treatment showing the strongest homogeneous selection dominance. Random processes (|βNTI| < 2) decreased compared to the CK treatment, while the L treatment significantly increased dispersal limitation. For the fungal communities, litter inputs enhanced both homogeneous selection and dispersal limitation, with the L and LR treatments enhancing homogeneous selection and the M treatment strengthening dispersal limitation. Both the bacterial and fungal communities under the LR treatment exhibited broader niche breadth (Figure 7).

### 3.5. Pathways in Which Different Subsurface Organ Litter Inputs Affect SOC

**Figure 8:**
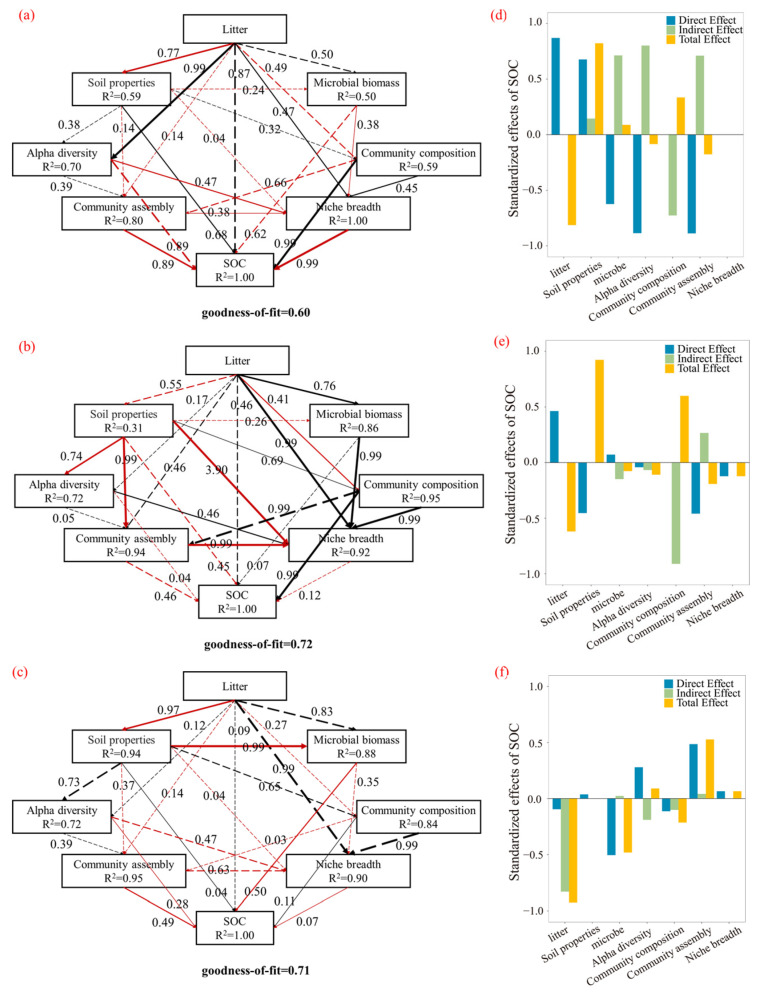
Direct and indirect relationships between litter decomposition dynamics (**a**) rhizome, (**b**) root, (**c**) mixed) and soil microbial biomass, community composition, assembly processes, niche breadth, physicochemical properties, and organic carbon. Standardized effects in SEM for (**d**) rhizome, (**e**) root, and (**f**) mixed litter. Red and black solid lines indicate significant negative and positive relationships, respectively; dashed lines denote non-significant path coefficients. Numbers adjacent to arrows represent standardized path coefficients, with arrow width corresponding to effect size.

Through analysis of the structure of environmental variables and microbial-related data, we aimed to reveal how litter decomposition affects soil organic carbon through direct and indirect pathways (Figure 8). The litter decomposition dynamics measured included metrics such as mass loss, decomposition rate, and the C/N ratio. The soil factors included pH and TN. Microbial biomass included T PLFA, B PLFA, and F PLFA. Bacterial and fungal diversity included the Chao1 and Shannon indices. Community assembly and niche breadth included βNTI dimensionality reduction data for bacteria and fungi, as well as the niche breadth of dominant species. Community composition included the dominant bacterial and fungal species [31]. Overall, the litter effects on SOC were mediated indirectly by shaping the microbial community structure/function and regulating the soil physicochemical properties, with distinct pathways driven by different belowground organ litters. The “high-quality” root litter indirectly regulated SOC by altering community composition. The “low-quality” rhizome litter predominantly mediated carbon accumulation through abiotic pathways. The synergistic effects of mixed litter reflect the resource complementarity in carbon sequestration.

## 4. Discussion

### 4.1. Litter Decomposition Characteristics

Current studies demonstrate that plant interactions show limited effects on litter decomposition, with litter quality and environmental conditions exerting greater influence. Heredia-Acuña specifically confirmed that root interactions between coexisting species did not affect root litter decomposition, eliminating plant–plant interactions as key determinants in this process [40]. Parallel findings by Wambsganss revealed minimal tree diversity effects in forest ecosystems, where initial litter quality and environmental factors more strongly affect fine root litter mass loss than species mixture effects [41]. We found that LR decomposed the fastest, while L decomposed the slowest, consistent with decomposition patterns of “high-quality” single-species and “low-quality” single-species litter in grassland ecosystems [42]. In this study, LR was classified as “high-quality” litter with a low C/N ratio, exhibiting characteristics more favorable for microbial decomposition. The physical structure promoted microbial colonization and enhanced internal organic matter decomposition, while the chemical composition contained higher proportions of labile components (simple sugars, proteins, and soluble organic compounds) [33], which rapidly supplied energy and nutrients to accelerate decomposition. Cai et al. proposed that intermittent flooding conditions provide both adequate oxygen and moisture for microbial metabolic activity while facilitating optimal litter–microbe contact, thereby creating favorable conditions for microbial survival and enhanced litter decomposition [43]. Our results similarly demonstrate that regardless of the litter addition method, both the litter mass loss and decomposition rates were highest under intermittent flooding conditions, consistent with these findings.

Hong et al. [44] found that “high-quality” litter more effectively promoted soil nitrogen retention, a pattern also observed in our study. Litter with a low C/N ratio likely facilitates greater carbon release during decomposition while retaining or converting more N, thereby altering litter nutrient content and stoichiometric characteristics [33]. Chen and Liu et al. proposed that intermittent flooding conditions might facilitate greater retention and transformation of carbon (C) and nitrogen (N) in decomposing litter [45,46], which contrasts with our findings. In this study, litter under non-flooded conditions exhibited higher C and N contents compared to both flooding regimes, potentially because the absence of water leaching allowed for more efficient nutrient retention within the litter matrix.

### 4.2. Litter Addition Alters Soil Organic Carbon Content and Physicochemical Properties

The M treatment resulted in a higher SOC content, whereas Zeng et al. [47] reported that “high-quality” single-species litter promoted greater SOC sequestration in grassland ecosystems, differing from our findings. In this study, the mixed litter addition exhibited synergistic effects on carbon input, leading to higher SOC accumulation compared to single litter additions, which partially diverged from our initial hypothesis (1). We observed the highest soil organic carbon content under non-flooded conditions, consistent with Bouma et al. [48], who reported increased carbon accumulation in non-flooded wetland soils. The well-aerated conditions in non-flooded soils support efficient aerobic microbial decomposition, facilitating carbon release from litter and its transformation into various carbon forms [49]. The initial soil pH averaged 6.88–7.05 (near-neutral range), while litter addition generally reduced the soil pH, with the lowest value reaching 6.52 (significantly more acidic). As the litter contained abundant organic components, microbial metabolic activity during decomposition played a key role. Through respiration and enzymatic reactions, microbes progressively converted macromolecular organic matter in litter into low-molecular-weight organic acids. The accumulation of these organic acids directly increased the hydrogen ion concentration in the soil, consequently lowering the soil pH [50].

### 4.3. Litter Addition Alters Soil Microbial Biomass

Numerous studies have employed PLFA analysis to assess soil microbial biomass and community composition [36]. While Bai et al. [51,52] observed that “high-quality” single-species litter preferentially enhanced bacterial growth in forest ecosystems, our study found the highest bacterial biomass under rhizome litter addition. This divergence may have arisen from rhizomes’ dual characteristics as slow-decomposing organs with a high C/N ratio and as belowground nutrient reservoirs, which sustain microbial proliferation through gradual carbon and nitrogen release [53]. The M treatment yielded the highest fungal biomass, consistent with the findings of Wan et al. [54], who reported that mixed litter inputs in forest ecosystems stimulated fungal growth. This effect is attributed to the rich content of complex carbon sources preferred by fungi, such as cellulose and hemicellulose, which enhance fungal growth and metabolic activity [55]. However, decomposition dynamics varied significantly across the litter types, and microbial biomass responses were not uniform. Litter compositional traits, particularly the C/N ratio, were observed to differentially influence microbial communities.

### 4.4. Litter Addition Modifies Microbial Survival Strategies and Community Assembly

Proteobacteria preferentially inhabit environments with high organic matter content and abundant labile carbon sources, exhibiting r-strategy characteristics that enable rapid response to resource inputs. In contrast, Acidobacteriota thrive in low-nutrient, carbon-limited soils, representing typical K-strategist microbial groups [23]. Dong et al. observed that Proteobacteria reached their highest relative abundance, while Acidobacteriota showed the lowest during mixed forest litter decomposition in forest ecosystems [56]. This contrasts with our findings, where the root litter (single litter type) treatment yielded the maximum Proteobacteria and minimum Acidobacteriota abundance in soils. This discrepancy may stem from differences in the litter decomposition rates. Dong’s study demonstrated significantly faster decomposition in mixed forest litter compared to monoculture litter, while our study found that roots decomposed most rapidly. Consequently, higher decomposition rates facilitate faster nutrient input into soils, particularly supporting the proliferation of r-strategist microbial groups, such as Proteobacteria. The root litter treatment showed the highest abundance of Ascomycota, while the mixed litter treatment yielded the maximum Basidiomycota abundance, consistent with Jing’s findings [57]. This likely reflects Ascomycota’s preference for nutrient-rich substrates, such as roots, whereas Basidiomycota thrive in mixed litter with moderate lignin content [58]. In contrast to LR, the L treatment was favored by K-strategist microbial communities and concurrently exhibited the lowest SOC content among all treatments. Under both LO and ME conditions, the addition of “high-quality” LR litter resulted in the highest bacterial and fungal diversity, whereas the “low-quality” L treatment yielded the lowest microbial diversity. The nutrient-rich composition of LR litter provided more abundant nutritional resources, stimulating the growth of diverse bacterial and fungal taxa, thereby maximizing microbial α-diversity and creating distinct community structures compared to other treatments. Wang et al. [59] observed that both high- and medium-quality litter additions enhanced microbial diversity in a stream ecosystem, aligning with our findings. Hydrological conditions played a pivotal role in regulating microbial community composition and distribution, as evidenced by our PCoA analysis, which demonstrated statistically significant flooding effects on both bacterial and fungal communities.

Litter addition significantly altered microbial interactions, disrupting the original ecological equilibrium in both bacterial and fungal communities and markedly enhancing microbial connectivity [60]. This shift occurred because litter inputs provided diverse carbon and nitrogen sources, along with other nutrients, driving microbial species to intensify their competitive and cooperative relationships during resource acquisition and utilization [61]. The LR treatment increased the average path length of bacterial networks, consistent with findings by Tanunchai et al. in forest ecosystems [62]. This indicates that LR litter addition enhanced the complexity of information transfer and material exchange pathways within bacterial communities. The relatively loose physical distribution of LR in the soil facilitated bacterial colonization, thereby modifying bacterial interaction patterns and resulting in more complex and diverse community structures and functions [33,60]. Furthermore, LR addition increased the modularity coefficient of fungal communities, a pattern similarly observed by Chen et al. [63] in wetland ecosystems. Higher modularity indicates the formation of more discrete, functionally specialized modules within fungal networks, where intra-module connections are dense, while inter-module linkages remain relatively weak [64]. This architecture promotes efficient resource utilization through coordinated metabolic activities while minimizing cross-module energy loss, thereby accelerating LR decomposition.

From a microbial dispersal perspective, species diffusion was significantly more active under the LR treatment, particularly in lacustrine wetlands, where roots from macrophytes (*P. australis*) enhance microbial transport, aligning with the findings of Dehlin et al. [65]. This enhanced mobility stems from LR’s low C/N ratio, creating favorable environmental conditions for microbial dispersal, including decomposition-generated pore structures and nutrient gradients that facilitate microbial movement and propagation through the soil matrix [31]. The increase in homogeneous selection reflects stronger environmental filtering, which supports bacterial taxa with similar niche requirements. Conversely, reduced dispersal limitation indicates enhanced microbial mobility, allowing freer cross-habitat migration [66]. A broader niche breadth enables microbes to exploit a wider range of environmental resources [67]. In this study, the LR-treated soils exhibited a broader microbial niche breadth, enabling the microbial community to utilize a wider resource spectrum. This resource plasticity maintained higher diversity during root litter decomposition despite periodic flooding disturbances in lacustrine wetlands. We quantified phylogenetic turnover among the litter treatments using βNTI, revealing that litter addition generally enhanced homogeneous selection while reducing dispersal limitation in both the bacterial and fungal communities. The LR treatment particularly strengthened homogeneous selection dominance, consistent with the findings of Ma et al. [68], who demonstrated that in lacustrine wetlands, “high-quality” litter modified the soil conditions, making deterministic environmental filtering the primary assembly driver and reducing stochasticity. Conversely, the “low-quality” L treatment increased dispersal limitation, likely due to its slower decomposition, which promoted the accumulation of dense organic layers. These layers reduced soil pore connectivity, physically restricting microbial dispersal and active migration [69]. This mechanistic constraint partially explains L’s comparatively lower SOC contribution relative to the other treatments in lacustrine settings, where carbon retention depends on microbial access to root channels. Consistent with Hypothesis (2), the “high-quality” litter shifted microbial communities toward r-strategists, reinforcing deterministic processes while reducing stochastic influences during community assembly.

### 4.5. Pathways of Different Litter Inputs Affecting SOC

The “high-quality” root litter indirectly regulated SOC by altering community composition (e.g., increasing the relative abundances of Proteobacteria and Ascomycota). Its rapid decomposition traits and nitrogen-induced homogeneous selection jointly enhanced microbial control over carbon transformation, manifested through increased fungal biomass and expanded niche breadth. This likely occurred as labile litter modifies microbial nutrient acquisition strategies, thereby driving structural community differences that ultimately alter carbon accumulation patterns [70]. In contrast, the “low-quality” rhizome litter predominantly mediated carbon accumulation through abiotic pathways. Its slow decomposition reduced the soil pH and increased the α-diversity, thereby enhancing the soil physicochemical effects on the SOC. He et al. similarly demonstrated that litter inputs primarily influence microbial communities by modifying soil properties [31], subsequently affecting carbon accumulation. The synergistic effects of mixed litter reflect the resource complementarity in carbon sequestration, both by improving microbial functionality to enhance metabolic and by alleviating nutrient limitations to promote SOC accumulation [71].

## 5. Conclusions

Root litter was found to decompose the fastest, while rhizome litter decomposed the slowest, with intermittent flooding being most conducive to litter decomposition. The mixed litter of roots and rhizomes exhibited synergistic effects on decomposition, demonstrating significantly greater impacts on lake wetland SOC compared to single-organ litter. The order of the SOC content under the different flooding regimes was as follows: non-flooded > intermittently flooded > permanently flooded. The addition of belowground plant litter significantly altered the soil microbial community composition and diversity while enhancing microbial interactions. The root litter addition selected for r-strategist microbes, increasing community complexity and stability. This treatment strengthened homogeneous selection, reduced dispersal limitation, and expanded niche breadth. In contrast, rhizome litter enhanced K-strategists and imposed stronger dispersal limitation on microbial communities. Belowground organ litters regulate soil organic carbon accumulation through distinct pathways, including root litter, via rapid decomposition, enriching r-strategist microbial groups, and enhancing homogeneous selection to promote SOC accumulation. Rhizome litter indirectly modulates SOC through abiotic pathways, such as acidification effects. Mixed rhizome–root litter boosts SOC accumulation by improving nutrient availability and strengthening microbial network complexity.

## Figures and Tables

**Table 1:** Changes in litter mass loss (R), decomposition rate (SL), litter C (C), litter N (N), and litter C/litter N (C/N) under different litter addition methods and flooding conditions. Significant differences among the same flooding conditions are indicated by uppercase letters (*p* < 0.05), while differences within the same flooding condition but different litter addition methods are indicated by lowercase letters (*p* < 0.05). LO indicates non-flooded, ME indicates intermittently flooded, HI indicates permanently flooded, L represents rhizomes, LR represents roots, and M represents a mixture of the two.

		R	SL	N (g/kg)	C (g/kg)	C/N
LO	L	0.3883 ± 0.0087 ^Cc^	0.0035 ± 0.0014 ^Cb^	6.7067 ± 0.1012 ^Ac^	498.5767 ± 1.5866 ^Aa^	74.3521 ± 1.1767 ^Aa^
	LR	0.5049 ± 0.0032 ^Ca^	0.0065 ± 0.0003 ^Ca^	20.3000 ± 0.0985 ^Aa^	418.5333 ± 3.9845 ^Ac^	20.6171 ± 0.0974 ^Ac^
	M	0.4344 ± 0.0025 ^Cb^	0.0044 ± 0.0005 ^Cab^	13.5450 ± 0.1830 ^Ab^	453.5733 ± 1.6321 ^Ab^	33.4895 ± 0.3479 ^Ab^
ME	L	0.5361 ± 0.0010 ^Ac^	0.0066 ± 0.0003 ^Ac^	7.1167 ± 0.1206 ^Ac^	471.8433 ± 24.3541 ^Aa^	66.2774 ± 2.3768 ^Aa^
	LR	0.6488 ± 0.0008 ^Aa^	0.0103 ± 0.0004 ^Aa^	19.3433 ± 0.3089 ^Aa^	450.0800 ± 3.1980 ^Aa^	23.2703 ± 0.2416 ^Ac^
	M	0.5850 ± 0.0033 ^Ab^	0.0088 ± 0.0004 ^Ab^	12.5367 ± 0.3048 ^Ab^	405.2383 ± 2.1059 ^Ab^	32.3377 ± 0.8448 ^Ab^
HI	L	0.4776 ± 0.0017 ^Bc^	0.0060 ± 0.0004 ^Bb^	8.5300 ± 0.3205 ^Ac^	441.1567 ± 4.9133 ^Ba^	51.7565 ± 1.5880 ^Aa^
	LR	0.5450 ± 0.0033 ^Ba^	0.0077 ± 0.0005 ^Ba^	10.6800 ± 0.2972 ^Aa^	167.6767 ± 0.5554 ^Bc^	15.7091 ± 0.4857 ^Ac^
	M	0.5156 ± 0.0035 ^Bb^	0.0067 ± 0.0007 ^Bab^	9.3067 ± 0.0978 ^Ab^	294.605 ± 5.6638 ^Bb^	31.6617 ± 0.9371 ^Ab^

**Table 2:** Changes in the soil pH, SOC, DOC, TN, and AP under different litter addition methods and flooding conditions. Significant differences among the same flooding conditions are indicated by uppercase letters (*p* < 0.05), while differences within the same flooding condition but different litter addition methods are indicated by lowercase letters (*p* < 0.05). LO indicates non-flooded, ME indicates intermittently flooded, HI indicates permanently flooded, CK represents the blank control without adding litter, L represents rhizomes, LR represents roots, and M represents a mixture of the two.

		pH	SOC (g/kg)	DOC (mg/kg)	TN (g/kg)	AP (mg/kg)
LO	CK	7.05 ± 0.05 ^Ba^	15.62 ± 0.41 ^Ad^	285.19 ± 11.98 ^Ad^	0.95 ± 0.03 ^Ac^	13.23 ± 0.61 ^Ac^
	L	6.56 ± 0.02 ^Bb^	18.39 ± 0.94 ^Ac^	315.55 ± 9.72 ^Ac^	1.62 ± 0.08 ^Aa^	15.62 ± 0.23 ^Ab^
	LR	6.52 ± 0.06 ^Bb^	23.20 ± 1.03 ^Ab^	351.16 ± 19.71 ^Ab^	1.72 ± 0.18 ^Aa^	16.37 ± 0.34 ^Aa^
	M	7.02 ± 0.06 ^Ba^	25.99 ± 2.78 ^Aa^	428.47 ± 14.12 ^Aa^	1.21 ± 0.05 ^Ab^	11.78 ± 0.44 ^Ad^
ME	CK	6.88 ± 0.05 ^Ab^	10.91 ± 0.29 ^Bd^	105.23 ± 3.84 ^Bd^	0.47 ± 0.04 ^Bc^	16.54 ± 0.18 ^Aa^
	L	7.03 ± 0.10 ^Aa^	11.82 ± 0.34 ^Bc^	118.21 ± 4.83 ^Bc^	0.59 ± 0.04 ^Ba^	9.01 ± 0.66 ^Ad^
	LR	6.92 ± 0.06 ^Aab^	13.09 ± 0.20 ^Bb^	135.58 ± 3.72 ^Bb^	0.60 ± 0.02 ^Ba^	12.85 ± 0.65 ^Ac^
	M	6.83 ± 0.09 ^Ab^	13.78 ± 0.22 ^Ba^	176.27 ± 2.00 ^Ba^	0.55 ± 0.01 ^Bb^	14.64 ± 0.35 ^Ab^
HI	CK	6.99 ± 0.05 ^Aa^	5.18 ± 0.15 ^Cd^	105.10 ± 2.12 ^Bd^	0.30 ± 0.02 ^Cc^	16.60 ± 0.33 ^Aa^
	L	6.99 ± 0.04 ^Aa^	7.11 ± 0.20 ^Cc^	123.28 ± 3.11 ^Bc^	0.40 ± 0.01 ^Cb^	10.46 ± 0.25 ^Ac^
	LR	6.88 ± 0.05 ^Ab^	7.86 ± 0.20 ^Cb^	165.94 ± 3.20 ^Ba^	0.45 ± 0.02 ^Ca^	8.51 ± 0.33 ^Ad^
	M	6.84 ± 0.02 ^Ab^	8.50 ± 0.24 ^Ca^	143.08 ± 1.72 ^Bb^	0.47 ± 0.02 ^Ca^	13.51 ± 0.19 ^Ab^

**Table 3:** Topological indices of soil bacterial and fungal community networks following different litter decomposition treatments. CK represents the blank control without adding litter, L represents rhizomes, LR represents roots, and M represents a mixture of the two.

	Litter Treatment	Node	Edge	Positive Link (%)	Negative Link (%)	Modularity	Average Clustering Coefficient	Average Degree	Average Path Distance
Bacteria	CK	391	7608	72.96	27.04	0.435	0.545	38.916	2.738
	L	427	12,358	61.74	38.26	0.462	0.584	57.883	2.464
	LR	347	9487	54.74	45.26	0.277	0.582	54.68	2.697
	M	376	14,883	51.19	48.81	0.296	0.648	79.165	2.266
Fungi	CK	184	2636	86.49	13.51	0.448	0.708	28.652	2.841
	L	211	2584	81.85	18.15	0.607	0.704	24.493	3.519
	LR	195	2278	68.53	31.47	0.491	0.594	23.364	2.928
	M	204	3310	92.48	7.52	0.542	0.735	32.451	2.787

## Data Availability

Data are contained within the article and the Supplementary Materials.

## Supplementary Materials

The following supporting information can be downloaded at: https://www.mdpi.com/article/10.3390/microorganisms13051146/s1, Figure S1. The soil temperature at which the litter decomposes in a year; Table S1. Statistical Normality Testing (Shapiro-Wilk) of Ecological Parameters in Heterogeneous Litter Decomposition Under Uniform Flooding Conditions; Table S2. Distributional Normality for Ecological Parameters in Standardized Litter Decomposition Under Variable Submersion Conditions (Shapiro-Wilk); Table S3. Analysis of Variance Homogeneity in Ecological Parameters of Heterogeneous Litter Decomposition Under Uniform Flooding Environments; Table S4. Analysis of Variance Homogeneity in Ecological Parameters of Litter Decomposition Across Differential Flooding Environments.
