# Supplementary material for: The Impact of Litter from Different Belowground Organs of Phragmites australis on Microbial-Mediated Soil Organic Carbon Accumulation in a Lacustrine Wetland
Source: Microorganisms. 2025 May 16;13(5):1146. doi: 10.3390/microorganisms13051146 (PMC12114342; doi:10.3390/microorganisms13051146)
Supplement: Supplementary file 1 [file microorganisms-13-01146-s001.zip › microorganisms-3613004-supplementary.pdf]

Supplementary Material

The impact of litter from different belowground organs of *Phragmites australis* on microbial-mediated soil organic carbon accumulation in a Lacustrine wetland

Chong Chen[1], Yong Wang[1, *], Liu Yang[1], Yongen Min[1], Keming Yue[1], Sitong Lu[1], Hongfeng Bian[1, *], Xue Wang[1], Leilei Zhang[1]

[1] Key Laboratory of Wetland Ecology and Vegetation Restoration, Ministry of Ecology and Environment, Northeast Normal University, Changchun 130117, China

* Corresponding author:

E-mail addresses: wangy833@nenu.edu.cn (Y. Wang); bianhf108@nenu.edu.cn (H.B.)

Postal address: 5268 Renmin Street, Changchun, Jilin Province, PRC

Figure S1. The soil temperature at which the litter decomposes in a year.

Table S1. Statistical Normality Testing (Shapiro-Wilk) of Ecological Parameters in Heterogeneous Litter Decomposition Under Uniform Flooding Conditions.

Table S2. Distributional Normality for Ecological Parameters in Standardized Litter Decomposition Under Variable Submersion Conditions (Shapiro-Wilk).

Table S3. Analysis of Variance Homogeneity in Ecological Parameters of Heterogeneous Litter Decomposition Under Uniform Flooding Environments.

Table S4. Analysis of Variance Homogeneity in Ecological Parameters of Litter Decomposition Across Differential Flooding Environments.

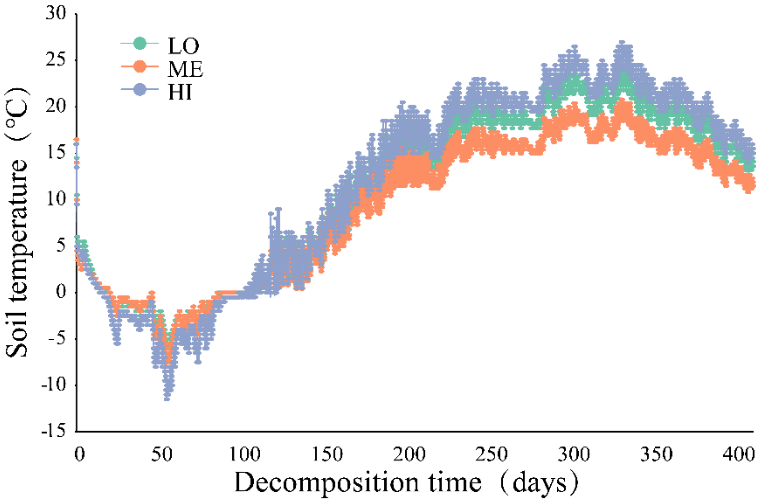

Figure S1. The soil temperature at which the litter decomposes in a year.

Table S1. Statistical Normality Testing (Shapiro-Wilk) of Ecological Parameters in Heterogeneous Litter Decomposition Under Uniform Flooding Conditions.

	R	SL	Litter-N	Litter-C	Litter-C/N	SOC	DOC	TN	AP	pH	TPLFA	BPLFA	FPLFA
L	0.460	0.056	0.081	0.078	0.130	0.102	0.061	0.058	0.627	0.062	0.055	0.064	0.059
LR	0.094	0.057	0.052	0.051	0.064	0.063	0.055	0.077	0.114	0.084	0.057	0.053	0.069
M	0.295	0.073	0.020	0.075	0.059	0.076	0.060	0.053	0.195	0.268	0.313	0.550	0.090

Table S2. Distributional Normality for Ecological Parameters in Standardized Litter Decomposition Under Variable Submersion Conditions (Shapiro-Wilk).

	R	SL	Litter-N	Litter-C	Litter-C/N	SOC	DOC	TN	AP	pH	TPLFA	BPLFA	FPLFA
LO	0.554	0.092	0.050	0.096	0.052	0.588	0.130	0.391	0.114	0.096	0.067	0.346	0.114
ME	0.142	0.050	0.064	0.210	0.051	0.312	0.055	0.103	0.238	0.608	0.417	0.499	0.053
HI	0.050	0.115	0.394	0.055	0.062	0.865	0.142	0.656	0.327	0.648	0.533	0.279	0.056

Table S3. Analysis of Variance Homogeneity in Ecological Parameters of Heterogeneous Litter Decomposition Under Uniform Flooding Environments.

	R	SL	Litter-N	Litter-C	Litter-C/N	SOC	DOC	TN	AP	pH	TPLFA	BPLFA	FPLFA
LO	0.025	0.050	0.241	0.240	0.084	0.045	0.076	0.082	0.087	0.136	0.279	0.192	0.467
ME	0.126	0.231	0.252	0.026	0.011	0.930	0.233	0.018	0.309	0.656	0.368	0.331	0.016
HI	0.052	0.561	0.268	0.069	0.050	0.880	0.890	0.701	0.080	0.169	0.197	0.588	0.088

Table S4. Analysis of Variance Homogeneity in Ecological Parameters of Litter Decomposition Across Differential Flooding Environments.

	R	SL	Litter-N	Litter-C	Litter-C/N	SOC	DOC	TN	AP	pH	TPLFA	BPLFA	FPLFA
water	0.044	0.217	0.007	0.002	0.002	0.000	0.018	0.000	0.638	0.000	0.000	0.000	0.061
